# Qualitative insights from patient/caregivers, and clinicians on routine use of the EQ-5D-Y-5L in clinical paediatric care—results from a pilot feasibility and acceptability trial

**DOI:** 10.1007/s11136-026-04202-2

**Published:** 2026-03-13

**Authors:** Renee Jones, Kathe Holmes, Nancy Devlin, Kim Dalziel, Harriet Hiscock

**Affiliations:** 1 Melbourne Health Economics, University of Melbourne, Victoria, Australia; 2 Health Services and Economics, Murdoch Children’s Research Institute, Level 10/305 Grattan St, Melbourne , VIC 3000 Australia; 3 Victorian Comprehensive Cancer Centre Alliance, Victoria, Australia; 4 Department of Paediatrics, The University of Melbourne, Victoria, Australia

**Keywords:** Patient reported outcome measures, PROMs, Clinical care, Implementation, Co-design, Pediatrics, Paediatrics, EQ-5D-Y-5L, Consumer involvement, Quality of life

## Abstract

**Aim:**

To qualitatively understand the experiences of patient/caregivers, and clinicians of piloting a generic Paediatric Patient Reported Outcome Measure (P-PROM), the EQ-5D-Y-5L, in Routine Outpatient Care for Kids (ROCK), known as the P-PROM ROCK Program.

**Methods:**

Semi-structured interviews and focus groups were conducted online and in-person between April-June 2024. Participants were eligible if they received (patient/caregiver) or delivered (clinician) the P-PROM ROCK Program in the pilot feasibility and acceptability randomised clinical trial. Interviews were audio recorded and transcribed. Detailed notes were generated for focus groups. Transcripts and notes were coded and summarised into themes using thematic analysis.

**Results:**

Nine interviews (n = 9 patient/caregivers) and two focus groups (n = 7 clinicians) were conducted. Participants shared that the simplicity and ease of the co-designed P-PROM ROCK Program (including EQ-5D-Y-5L, personalised PROMpt, patient and clinician resources, and integration with systems) was critical to completion and use of EQ-5D-Y-5L in clinical care. When P-PROM results were discussed in appointments, participants shared how this improved communication, resulted in more holistic care, and provided parents with new perspectives on their child’s health. P-PROM results were not always discussed, and participants suggested reminder systems to minimise this. Additionally, short appointments and highly specialised clinical scope were barriers to holistic care. Finally, participants found the intervention too brief and wanted longer to better understand its benefits and limitations.

**Conclusion:**

The simplicity and ease of P-PROM programs are essential to their uptake and impact in clinical care. The P-PROM ROCK Program requires further refinements and piloting to truly understand its impact.

**Trial registration:**

ISRCTN16030620, https://www.isrctn.com/ISRCTN16030620.

**Supplementary Information:**

The online version contains supplementary material available at 10.1007/s11136-026-04202-2.

## Abstract (plain language summary)

Child health questionnaires ask about a child’s overall health and wellbeing. These questionnaires can sometimes be called ‘paediatric patient reported outcome measures’ or ‘health-related quality of life questionnaires’. They can be useful in clinical care as they provide doctors and nurses with information about how a child’s overall health is going from the patient’s view. Working with patient/caregivers and clinicians, we designed a way to use one of these questionnaires in routine clinical care. We then conducted a study to understand if asking patients to complete this questionnaire was acceptable and if it helped to improve the care they received. The point of this study was to talk with the children, parents and clinicians who took part in the study to understand their experiences. We found that it is acceptable to include these questionnaires in clinical care, especially when the questionnaire is simple and there are easy to understand resources available for families and clinicians. We also found that these questionnaires can help improve clinical care by making it more holistic. However, this is only possible when clinicians discuss the questionnaire responses with the family, which can sometimes be tricky when they are running short on time.

**Supplementary Information:**

The online version contains supplementary material available at 10.1007/s11136-026-04202-2.

## Introduction

Paediatric Patient Reported Outcome Measures (P-PROMs) are tools used to capture a child’s overall health and quality of life from their own or their caregiver’s perspective [1, 2]. Historically, P-PROMs have been used in clinical trials, population research, and patient registries [3–5]. More recently, they are being used in routine clinical care as a mechanism to enable more patient-centred care [3, 6]. Despite the growing use of PROMs in adult clinical care, their uptake in paediatric care remains low [6, 7]. Whilst PROMS are relatively easy to measure they are challenging to embed in routine health care delivery [8]. Their implementation requires behaviour change which can be supported through evidence-based implementation science frameworks [9, 10]. The provision of paediatric care differs significantly to that of adult care, hence further evidence on the use of P-PROMs in paediatric care is required.

To date, only eight studies have examined the quantitative impact of implementing a P-PROM in routine paediatric care [6, 11]. This initial quantitative research indicates that P-PROMs may improve the quality of care provided to children as well as improving the health related quality of life (HRQoL) of children [6]. However, there is limited qualitative evidence from users (including patients and clinicians) regarding their experiences of using P-PROMs in clinical paediatric care. Such qualitative evidence is essential to identify outcomes, both positive and negative, of implementation, as well as providing explanations for quantitative results. The authors were only able to identify two such qualitative studies [11, 12]. One study looked at clinician perspectives, finding beliefs (i.e., buy-in and burden); administration (i.e., P-PROM collection, scoring and displaying); clinical workflows; and incentives, were key factors impacting implementation in paediatric clinical contexts [12]. The other was a mixed-methods study that assessed patient and caregiver perspectives of the Dutch KLIK P-PROM program. This study found that although the P-PROM provided valuable insights into their child, it sometimes lacked importance, was repetitive, and was not always discussed by clinicians in their appointment [11].

This study is the first of its kind to report the qualitative perspectives of patient/caregivers, and clinicians, in parallel, regarding the implementation of a co-designed P-PROM program (P-PROM ROCK Program) across a range of medical disciplines—medical, surgical and behavioural. Capturing parallel perspectives across a range of disciplines is essential to ensure a thorough understanding is obtained regarding how the program is working or not working for different stakeholders in different clinical contexts. The P-PROM ROCK Program was co-designed with patients, caregivers and clinicians to inform how the EQ-5D-Y-5L [13], a 5-item generic P-PROM, would be implemented and used in routine paediatric outpatient care, to maximise positive impact on patient-clinician visits [14, 15]. Routine outpatient care is where speciality care is provided to children by specialised clinicians, often occurring via hospital-based office visits. The P-PROM ROCK Program was co-designed for use in this context given the benefits of PROMs in chronic care management [16].

The aim of this study is to qualitatively understand the experiences of patient/caregivers, and clinicians in parallel regarding the piloting of a generic P-PROM implemented via the PROM ROCK Program. More specifically, to explore 1) experiences with the different co-designed elements of the P-PROM ROCK Program, 2) experiences regarding the impact of the P-PROM ROCK Program on clinical care, and 3) perspectives on the future potential of the P-PROM ROCK Program [14, 15].

## Methods

### Study design and setting

This qualitative study used semi-structured interviews and focus groups. The Consolidated Criteria for Reporting Qualitative Research (COREQ) guidelines were followed for reporting [17].

This study is Phase 4 of a larger co-design and implementation project, known as the P-PROM ROCK study. In Phases 1 and 2, the P-PROM ROCK Program was co-designed and found to be suitable for implementation [14, 15]. In Phase 3, a pilot feasibility and acceptability randomised control trial (RCT) was conducted (P-PROM ROCK Program was intervention and standard care was control), and quantitative results were reported [18]. In this Phase 4 study, the qualitative results from the pilot feasibility and acceptability RCT are reported. The pilot feasibility and acceptability RCT was conducted in four outpatient clinics for children aged 4–17 years (complex asthma, behavioural sleep problems, encopresis, and chronic constipation) at The Royal Children’s Hospital (RCH), Melbourne Australia. These four clinics were selected as it was assessed that a generic PROM may highlight different types of information in each clinic that would be unique from the condition-specific information already captured. These same four clinics were included in prior co-design phases of this research [14, 15]. Outpatient appointments are specialised hospital-based clinician-patient visits. To receive specialist care in one of these clinics, patients must be referred from their primary care provider and meet referral criteria. This study received ethics approval (HREC/103264/RCHM-2024) and was prospectively registered with ISRCTN (ISRCTN16030620).

The co-designed P-PROM ROCK Program supports how the EQ-5D-Y-5L is integrated and used in routine paediatric clinical outpatient care. The validity of the EQ-5D-Y-5L in similar paediatrics patients and setting has been previously reported [19]. The EQ-5D-Y-5L (Australian English version) was completed by patients or their caregivers in the intervention arm of the pilot feasibility and acceptability RCT. The EQ-5D-Y-5L is a generic P-PROM consisting of five items (mobility, looking after myself, doing usual activities, having pain or discomfort, feeling worried sad or unhappy) with each item having five responses levels, and the EQ visual analogue scale (EQ VAS), which asks patients to report their overall health on a scale of 0–100 [10]. Patients were sent a copy of the P-PROM and information about participation in the study by the research team who also followed up the P-PROM completion. After completing the EQ-5D-Y-5L, patients were asked to identify which EQ-5D-Y-5L items they wanted to discuss with their clinician. The EQ-5D-Y-5L was self-reported for patients eight years or older who were considered able to self-report by their caregiver, with other responses (i.e. younger than 8 years or considered unable to complete) proxy reported. The EQ-5D-Y-5L was chosen for its brevity [16], psychometric performance [19], and availability of funding to support implementation.

The co-designed P-PROM ROCK program consists of six key elements (1) clinician education (60-min group training), (2) patient/caregiver introduction to P-PROM (information flyer and video), (3) unique system of scoring, displaying of P-PROM results in the patient's EMR (EQ-5D-Y-5L results displayed in table format and additional patient PROMpt question where patients are asked to select which EQ-5D-Y-5L item(s) they want to discuss with clinician is highlighted in red and yellow), (4) integration into patient journey, clinical workflow and clinical systems (P-PROM attached to appointment, patient can complete P-PROM up to seven days before appointment online via the patient portal (EPIC MyChart) or on paper in the waiting room, results displayed in EMR), (5) supports for patients and caregivers after completing P-PROM (2-page practical guide with suggestions on where to seek support if concerned about a P-PROM area (e.g., mobility concerns links to physiotherapy)), and (6) supports for clinicians to act on P-PROM results (1-page clinician decision support tool and 1-page resource for addressing urgent P-PROM concerns). Further details on the P-PROM ROCK Program are detailed in the co-design manuscript [14].

### Participants

Participants were eligible if they were involved in either receiving (patient/caregiver) or delivering (clinicians) the intervention arm of the P-PROM ROCK Program pilot feasibility and acceptability RCT. Eligible participants included adolescent patients aged 12–18 years and/or caregivers of patients aged 4–18 years, and clinicians including doctors, nurses, or allied health staff delivering the intervention.

To recruit patient/caregiver participants, members of the research team contacted eligible participants via phone or email who had agreed to be contacted for an interview. Eligible clinicians were invited via email to participate in a focus group. The research team purposively recruited to ensure a spread of participants across the four outpatient clinics.

### Data collection

Interviews and focus groups were conducted between April and June 2024, approximately 1–2-months after participant exposure to the P-PROM ROCK Program.

Nine qualitative interviews were conducted with patient/caregivers (two of the participants were adolescents accompanied by their caregivers as per their request). Interviews were approximately 30-min long and primarily conducted online via video conferencing (n = 8), however, where preferred, interviews were conducted in person (n = 1). Interviews were semi-structured and followed an interview guide (see supplementary materials) covering the following topics – experience completing EQ-5D-Y-5L, perceived impact on care, and views on resources and supports. During the interview, participants were shown copies of the EQ-5D-Y-5L and P-PROM ROCK resources/supports to prompt their memory. RJ, a female PhD student who was leading this study, conducted all interviews. RJ has a Master of Public Health, is not a parent or clinician, was not known to any of the interviewees, and is experienced in qualitative research. All interviews were audio recorded and subsequently transcribed.

Two focus groups were conducted with clinicians – one with five clinicians from the complex asthma outpatient clinic and the other with two clinicians who work across both the sleep and encopresis outpatient clinics. Focus groups were approximately 60-min long and conducted online via video conferencing (n = 1) or in person (n = 1), depending on clinician preference. Focus groups followed a discussion guide (see supplementary materials) covering the following topics – P-PROM ROCK Program likes and dislikes, impact on care, mechanisms for being useful or not useful, impact on role as clinician, harms and risks, and improvements for future. RJ facilitated the focus groups, and a research assistant was present to take notes. RJ was known to some of the focus group clinicians. All focus groups were recorded. Recordings and initial notes were reviewed by members of the research team, generating more detailed notes with participant quotes.

Participant numbers for inclusion in interviews and focus groups were not designed or decided based on a goal of data saturation, instead they were predicated on practical resourcing and anticipated numbers required to obtain a spread of views [21].

Table 1 outlines the participants characteristics.

**Table 1:** Participant characteristics

Characteristic	N (%) or mean (sd)
*Interviews*	
Completed patient/caregiver interview, N (% of interviews complete)	9* (100)
Affiliated outpatient clinic, N (% of interviews complete) Asthma Sleep Encopresis Colorectal surgery	3 (33.3) 2 (22.2) 2 (22.2) 2 (22.2)
Patient/caregiver gender, N (% of caregivers) Female	9 (100)
Caregiver age, mean (sd) of caregivers	42.1 (2.5)
Child age, mean (sd) of all children**	7.9 (2.4)
*Focus groups*	
Focus groups completed, N (% of focus groups)	2 (100)
Clinicians who took part in focus group, N (% of focus group participants)	7 (100)
Clinician type, N (% of focus group participants) Nurse Doctor	2 (28.6) 5 (71.4)
Affiliated outpatient clinic, N (% of focus group participants)** Asthma Sleep Encopresis Colorectal surgery	5 (71.4) 2 (28.6) 2 (28.6) 0

^*^Two of the participants were adolescents accompanied by their caregivers as per their request

^**^Including adolescent patients and children whose caregiver took part

^***^Clinicians can service multiple clinics (two clinicians serviced both the sleep and encopresis clinics)

### Data analysis

Interview and focus group data were analysed using thematic analysis [22]. Transcripts and detailed notes were read, coded, and clustered into themes [22]. Data were deductively coded using interview and focus group guides and inductively coded as additional coded as additional categories appeared. Coding was conducted independently by RJ and KH using NVivo 15.0. KH is a paediatric clinician, a parent, has training in qualitative research, and is not known to any participants. Once a final coding framework was established, RJ developed overarching themes with feedback and input from KH. Reflexivity was supported through regular meetings between KH and RJ to critique and appraisal of their approaches and application of codes [23]. A meeting between all authors was held after development of codes and application of framework to reflect, critique and appraise the research process [23]. Authors approached the analysis from health economics, health services research, and paediatric clinical orientations where the value of generic P-PROMs is considered possible and able to be evaluated. Once finalised by the study team, RJ applied this final thematic structure to all data. Saturation of themes was an explicit goal which was defined as the point in the coding process when the further analysis of data failed to identify any new codes [23].

A formal sub-group analysis was not conducted. Nonetheless, if themes were determined by coders to only relate to a certain participant type (patient, caregiver, clinician), this was specified in the results.

## Results

### Key themes

Table 2 summarises key themes and sub-themes. These are described in detail below and presented alongside key quotes (Tables 3, 4, 5).

**Table 2:** Summary of Theme and Sub-themes

Theme	Sub-theme
1. Simplicity and engagement	1.1 Engagement and trust are essential to P-PROM completion 1.2 EQ-5D-Y-5Lwas easy and simple to complete and use 1.3 Highlighting concerns (patient PROMpt) to prioritise discussion 1.4 Resources were essential and easy to access. EMR integration was essential
2. P-PROM impacts	2.1 Time and workflow 2.2 Importance of acknowledging and discussing P-PROM in appointment 2.3 Provision of more holistic care and new perspectives—benefits and challenges 2.4 Improved communication and satisfaction
3. Looking to the future	3.1 Longer and larger trial with tracking over time 3.2 Sustainability in current system

**Table 3:** Key Quotes for Theme 1—Simplicity and engagement

Sub-Theme	Quotes
1.1 Engagement and trust are essential to P-PROM completion	1) *“At the end of the day it’s for my child’s … health and well-being. I’d be happy to answer to anything really”* (Caregiver, Asthma, Pt 1) 2) *“Just knowing that it’s from the hospital and the hospital wants you to answer.”* (Caregiver, Asthma, Pt 1) 3) *“While I was filling it in, I wasn’t sure how it was going to help with the upcoming appointment”* (Caregiver, Encopresis, Pt 84) 4) *“If it said, ‘This will help your child’s blah blah blah,” because otherwise, yes, I want to help…but I have other things I’m trying to deal with.”* (Caregiver, Constipation, Pt 215) 5) *“It was kind of fun”* (Patient, Encopresis, Pt 80) 6) *“I just think you won’t be able to capture people’s attention if they’re borderline going to do it or not, and it looks a bit like… boring… Whereas if it had a couple of … characters on it and stuff.”* (Caregiver, Constipation, pt 215)
1.2 EQ-5D-Y-5L was easy and simple to complete and use in care	7) *“Easy”* (Patient, Asthma, Pt 7) 8) *“It's short and sweet… enjoyed the fact that it was short and straight to the point and there wasn't too much to go through”* (Caregiver, Asthma, Pt 7) 9) *“Straightforward, so I thought that was really good”* (Caregiver, Constipation, Pt 215) 10) *"There is something good about how simple it was" (Clinician (Doctor), Asthma)* 11) *“The questions were simple and straightforward. There's various questionnaires I use in other clinics that are long and there's lots of results to look at… it takes time to figure out…This was straightforward to interpret the results” (Clinician (Doctor), Sleep and Encopresis)*
1.3 Highlighting concerns (patient PROMpt) to prioritise discussion	12) *“I just liked that you could say if you wanted to talk to the doctor about those things.”* (Patient, Encopresis, Pt 80) 13) *“It was just good to remind me of the things that we went through, and to give me, again, a sense of agency that yes, I’ve answered your questions, but this is a priority for me or not a priority for me.”* (Caregiver, Constipation, Pt 215) 14) *“I don’t want to take the whole consultation up talking about everything, because most of it was quite simple… just the ones that I’ve ticked.”* (Caregiver, Constipation, Pt 219)
1.4 Resources were essential and easy to access. EMR integration was essential	15) *“They had brief information but then also if you were struggling in a certain area, there was then web pages and a resource you could access if that information wasn’t enough. That was good in the sense that if you didn’t want a lot of information, there was just the page there, but then if you needed a bit more there was the option to dive more into the topic.”* (Caregiver, Asthma, Pt 118) 16) *“I think the way you have designed it has offset other things, like you get worried as someone in an asthma clinic that you are going to have to try and deal with someone is having mobility issues or other things that we don't have the skills to deal with. But then I think having all the resources there and the patients having all the resources there, meant that actually I wasn't stressed about that, like that was going to be ok."* (Clinician (Doctor), Asthma) 17) *“I liked the prompts of how to respond to it. I like the little script. It wasn't long. That was good. But that was handy and getting used to it, having a little "Here's how to respond to it”* (Clinician (Doctor), Sleep and Encopresis) 18) *“EPIC is a complicated beast…And so it's not necessarily intuitive where to look….That sort of sense of being able to see this has not been filled in would have been useful”* (Clinician (Doctor), Sleep and Encopresis) 19) *"I don't know how it can kind of remind you at some point, whether it is at the start or end of the appointment, or when you open the EPIC file… Particularly it should be flagged if there is a problem."* (Clinician (Doctor), Asthma) 20) *“I guess if there was something there that, a couple of dot points that you’re concerned about, that would flag with them before the end of your appointment, that potentially that’s what you were hoping to have a quick chat about, would perhaps be beneficial.”* (Caregiver, Asthma, Pt 118)

**Table 4:** Key Quotes for Theme 2—P-PROM impacts

Sub-Theme	Quotes
2.1 Impact on time and workflow are critical for acceptability	21) *"I don't think it really impacted much (referring to clinic time)"* (Clinician (Doctor), Asthma) 22) *"I think when they were really good [referring to P-PROM result] and so you saw right at the start that they were competing fine, it kind of made my clinic quicker."* (Clinician (Doctor), Asthma)
2.2 Importance of acknowledging and discussing P-PROM results in appointment	23) *“We answered a lot of questions before his appointment, then his doctor actually asked about them… And she said, okay, I noticed that you had answered this. So, she’s read through it and asked him then how he was feeling that day… I thought that it was really good.”* (Caregiver, Sleep, Pt 105) 24) *“The minimum you want to do is acknowledge that they did that and you saw it and like flag it for next time, even if you don't have time to address it all"* (Clinician (Doctor), Asthma)
2.3 Provision of more holistic care and new insights – benefits and challenges	25) *“That whole approach, that, ‘everything about your kid matters.’”* (Caregiver, Encopresis, Pt 84) 26) “*I think it opened up this conversation about this other stuff that was going on and then trying to address that for them”* (Clinician (Doctor), Sleep and Encopresis) 27) “*You don't tend to ask, you tend to focus on the asthma. But it [the P-PROM] did help give you [with] an overall holistic idea of how they are travelling, which is helpful… which you don't usually stop and think to do in the middle of an asthma appointment”* (Clinician (Nurse), Asthma) 28) *“I feel like where these questionnaires really do make it a whole person approach, they [clinicians] really don't have the time. If they did, like they’d probably find other things that were attributing to all of this stuff”* (Caregiver, Encopresis, Pt 84) 29) *“I think they have to be more specific questions to each individual area of expertise, so whether it’s toileting, there needs to be more toileting questions”* (Caregiver, Encopresis, Pt 84) 30) *“Previous to this, I would've said it's much better to use an asthma specific one, but then I think in a dedicated asthma clinic you are always going to ask the questions that are in the asthma questionnaire anyway. I think there is value to people using them in between clinic visits, but I find it less helpful for them to do it [the asthma specific PROM] on the day of the appointment because I am going to ask them all of the same questions as part of my assessment. So having a general health questionnaire which I think is probably going to fluctuate with asthma control, yeh, I'm pretty happy with.”* (Clinician (Doctor), Asthma) 31) *“Me asking him [child] the questions helped me understand how he was feeling at that time. Where normally I might not ask him all of those questions.”* (Caregiver, Sleep, Pt 105) 32) *“I haven’t answered those questions before. It’s the first time, ‘Oh, she’s [child] feeling worried.’ It made me question herself, ‘Is she feeling worried because she can’t go to the toilet or school’ … It made me question more…. Maybe you are upset and don’t want to go to school because you can’t go to the toilet properly. So that helped to understand her feelings a little bit more.”* (Caregiver, Constipation, Pt 219)
2.4 Improved communication and satisfaction	33) “*The good impacts were that it gave me another reference point for communication and I guess it is another opportunity for the patient to be heard and voice their concerns.”* (Clinician, Asthma) 34) “*There was a sense of that interest in that overall well-being. And I guess this [referring to the P-PROM] gave a little bit of structure and a way to look at it that we don't necessarily have,.. in our routine."* (Clinician, Sleep and Encopresis) 35) “*It gives a bit more satisfaction about the care you are providing, assuming that the parents are satisfied too"* (Clinician, Asthma)

**Table 5:** Key quotes for Theme 3 – Looking to the future

Sub-Theme	Quotes
3.1. Longer and larger trial with tracking over time	36) *"Between us we all only had a handful in the intervention arm… I would be intrigued to trial it for longer and in more patients"* (Clinician (Doctor), Asthma) 37) *“I don’t really think it positively or negatively impacted her care at the time, because there wasn’t much on there that flagged anything that needed to be discussed … That’s not to say that if we were to do it for another appointment things may have been different” (Caregiver, Asthma, Pt 118)* 38) *“For example, having someone with a lower score and then watch it and see it progress would be more helpful… because you only see a person once in that [trial] you don't actually have a follow-up yet.” (Clinician (Nurse), asthma)* 39) *“See the improvements or, if it’s getting worse.” (Caregiver, Asthma, Pt 1)*
3.2. Sustainability in current system	40) *“I think I was always most worried about what are we going to do in the mental health space because we just don't have … a very functional system right now."* (Clinician (Doctor), Sleep and Encopresis) 41) *“I wonder if repeated use of this … whether it's a burden to be always going…they're sad and unhappy still and worried and still and we've not got on top of this. … I don't know if there's a potential harm there."* (Clinician (Doctor), Sleep and Encopresis)

#### Theme 1—Simplicity and engagement

##### Engagement and trust are essential to P-PROM completion

It was clear to some patient/caregivers how the P-PROM would help their child, and this (quote 1), as well as being asked by an organisation they trust (quote 2), meant they were engaged in completing the P-PROM. Others felt the benefit to their child was not obvious (quote 3) and thought better explaining this would improve engagement and completion (quote 4).

Whilst some patients found completing the P-PROM fun (quote 5), some caregivers worried it was a bit boring and felt that engagement might be higher if it was more appealing through use of drawings or characters (quote 6).

##### EQ-5D-Y-5L was easy and simple to complete and use

Almost all patient/caregivers and all clinicians found the EQ-5D--Y-5L quick, simple and easy to complete (quotes 7-9), meaning they were happy to complete it. Clinicians found it was simple to interpret and explain to patients (quotes 10-11).

##### Highlighting concerns (patient PROMpt) to prioritise discussion

Regarding the P-PROM ROCK patient PROMpt, where patients were asked via a single question to highlight which P-PROM item(s) they wanted to discuss with the clinician, patient/caregivers reported enjoying the agency and voice it provided them in terms of what was raised for discussion in their appointment (quotes 12-13). Caregivers and clinicians shared that it helped target the conversation and avoided taking up appointment time talking about irrelevant things (quote 14).

##### Resources were essential and easy to access. EMR integration was essential.

Patient/caregivers liked the brevity and practicality of the resources they received, finding they could still obtain further information if they needed by clicking links embedded (quote 15).

Clinicians felt that the resources received by families helped relieve the pressure on them to deal directly with identified issues they considered outside of their scope (quote 16).

When a patient had completed the P-PROM, it was intuitive for clinicians to see the P-PROM response in the EMR. Clinicians suggested it would be helpful if there was a flag in the EMR to indicate if the patient had completed the P-PROM or not (quote 18). Clinicians also shared concerns about forgetting to discuss the P-PROM result and suggested that an EMR reminder would be helpful (quote 19). Caregivers suggested a similar reminder system (quote 20).

The perception of the online portal was varied. While some patient/caregivers who completed the P-PROM online via the patient portal (the patient facing side of EMR), reported finding it easy to navigate others reported challenges accessing the resources via the portal.

#### Theme 2—P-PROM impacts

##### Time and workflow

Clinicians felt that overall, the P-PROM ROCK Program did not impact their clinic time negatively (quote 21). In fact, when the completed P-PROM identified patients as being very well, a clinician and patient both shared that this made the clinic appointment quicker (quote 22). Despite this, there were individual examples of discussing P-PROM results in appointments taking considerable time in clinic.

##### Importance of acknowledging and discussing P-PROM results in the appointment

Both caregivers and clinicians reported that for the P-PROM to impact clinical care, it was essential that results were reviewed by the clinician and discussed in the appointment. In some cases, this did not happen, and in other cases, caregivers reported that the clinician reviewed and responded to the P-PROM resulting in positive impacts for their care (quote 23). Clinicians shared that they should acknowledge that the patient had completed the P-PROM, even if they don’t have time to discuss the results (quote 24).

##### Provision of more holistic care and new insights – benefits and challenges

Patient/caregivers, and clinicians shared that one of the main ways the P-PROM impacted clinical care was by making care more holistic (quotes 25-26). They felt this was particularly important in the context of specialty outpatient care where care was often highly specific to a certain medical condition and often lacked consideration about the child’s broader wellbeing (quotes 27).

Both patient/caregivers and clinicians felt that clinicians did not always have time to address these broader concerns in speciality clinics (quotes 28). Another challenge of using the generic P-PROM within a specialty consultation was that some caregivers felt the generic P-PROM was too broad and wanted more questions specific to their child’s condition (quotes 29). In contrast, one clinician shared that the generic P-PROM provided them with new information, whereas information from condition specific P-PROMs would likely already be covered in current appointment discussions (quote 30).

Caregivers shared that by them or their child completing the P-PROM, they were able to gain new insights into how their child was going which they found helpful for understanding and managing their child’s condition (quotes 31-32).

##### Improved communication and satisfaction

Participants felt that the P-PROM provided patients with a structured way to have a voice in the clinical appointment which helped improve communication between patient and clinician (quote 33). Caregivers and clinicians felt that this improved communication and interest in the child’s broader wellbeing improved satisfaction with care (quotes 34-35).

#### Theme 3—Looking to the future

##### Longer and larger trial with tracking over time

Participants felt that in the trial they only got a limited window into what it would be like to use the P-PROM in care and were interested to trial it for longer and in more patients to better understand impacts (quotes 36-37). For example, despite not feeling as though the P-PROM ROCK Program impacted clinic time negatively in the trial, clinic time was still something clinicians were worried about if the program was to continue. Additionally, a caregiver also shared that they did not see any impact on clinical care in the trial as their child was well, but this might change if the P-PROM was used again in a future appointment (quote 37). Participants shared that a benefit of doing the P-PROM ROCK Program continuously in future would be the chance to gain additional insights by tracking and monitoring a child’s overall health over time (quotes 38-39).

##### Sustainability in current system

The studies specialist clinicians worried that some external community health systems where they might refer patients (to address P-PROM concerns), might not be adequate. For example, they worried community mental health programs were not currently well functioning which might be a potential risk if concerns remain unmet over a period of time (quotes 40-41).

## Discussion

This study is the first to explore the experiences of patient/caregivers, and clinicians, in parallel, of the use of a co-designed P-PROM program in paediatric outpatient clinical care. Patient/caregivers and clinicians highlighted that the ease and simplicity of the different co-designed elements of P-PROM ROCK Program were key to its acceptability and feasibility. They also shared that when the P-PROM was discussed in clinical care, this often resulted in more holistic care, improved patient-clinician communication and satisfaction with care. However, P-PROM results were not always discussed in appointments, which was frustrating for some families. Additionally, although more holistic care was seen as having benefits, many caregivers and clinicians acknowledged the challenges of providing more holistic care in time limited and resource constrained systems. Finally, patient/caregivers, and clinicians shared the benefits and potential risks of continuing the P-PROM ROCK Program, highlighting that the ability to track a child’s health over time would be beneficial for clinical care, however, doing this without the ability to appropriately respond to P-PROM concerns may result in unmet expectations and frustrations from families.

This is only the third study to qualitatively explore the perspectives of using a P-PROM program in paediatric clinical care. Contributing to this very limited qualitative evidence base is essential given the infancy of P-PROM programs [24, 25]. Furthermore, it is the only study to explore the perspectives of patient/caregivers, and clinicians in parallel, across a range of medical disciplines (medical, surgical and behavioural).

Like previous adult research [26] this study identified that the simplicity, ease and engagement were essential to P-PROM completion and use in clinical care. All participants shared these views. Whilst clinician acceptability was high in the study, implementation science tells us that ‘people-driven change’ is likely when the right support and systems are embedded [9]. We learnt that administrative support (to engage families and ensure PROM completion), EMR prompts (prompting patients and clinicians), and dedicated training/education were critical for P-PROM Rock program implementation and would be required for ongoing success.

Some feedback highlighted ways in which workflows and systems could be improved to make things even easier, including reminder systems for patients and clinicians to ensure P-PROM results were discussed in appointments, and more intuitive ways for patients to see affiliated resources and for clinicians to see if patients had not completed the P-PROM. These findings are consistent with other PROM and PREM literature suggesting that minimal burden and integration with workflows improves the likely success of such a program [8, 11, 12, 27, 28]. Additionally, patient/caregivers thought that adding characters or drawings would help improve P-PROM engagement. This same suggestion was made during co-design, however, was not possible in current EMR systems [14]. As systems improve, and the use of pictorials in P-PROMs becomes more common [29], this may be possible in future. Critically, these findings highlight the importance of co-design to the subsequent acceptability of an intervention [14].

These Phase 4 qualitative findings can help to better understand the quantitative trial results identified in Phase 3. [18] Clinicians in this study reported P-PROMs had minimal impact on the overall timing of clinics during the trial. However, in the quantitative survey completed as part of the trial, clinicians reported the inclusion of the P-PROM in clinical care added 5–10 min to appointment times [18]. This difference between quantitative and qualitative results could arise if, when completing the survey, clinicians only considered how much time the P-PROM added when there were extensive P-PROM concerns to discuss. Other research has demonstrated integrating P-PROMs into clinical care either reduces [30, 31], or have no overall impact on clinic time [32, 33]. Additionally, similar to other P-PROM research [11], caregivers in this study reported that completing the P-PROM as part of their child’s clinical care provided them with a better understanding of their child’s health. This outcome was not captured in the quantitative part of the trial [18], highlighting the importance of conducting the qualitative study. These findings also emphasise how perspectives and experiences can differ across stakeholders (i.e., impact on workflow important for clinicians and new insights important for caregivers), highlighting the importance of including stakeholder groups in parallel.

When considering scaling up the P-PROM ROCK Program or other similar programs, it is essential to balance the concerns of stakeholders with resourcing. System-level enablers were important in this study and likely critical for the successful implementation of P-PROMs and included embedding within the EMR, prompts within the EMR and clinician training and resources. Our co-designed process and resources could provide a helpful starting point for others seeking to implement generic P-PROMs. Participants raised concerns that hospital speciality clinic systems are designed and funded in a way that means clinicians only have short appointment times to address highly specific medical concerns. This limits the ability of clinicians to address wider health issues identified by P-PROM and provide truly holistic care. Other health systems in Australia have overcome this barrier through integration of a wellbeing coordinator who is responsible for linking families with appropriate community supports.[35] Additionally, the inclusion of generic P-PROMs and hence the provision of more holistic care in speciality settings often relies on well-functioning health systems outside the hospital to ensure patients can be managed by appropriate community supports. Participants in this study shared concerns about the functionality (i.e. availability, accessibility) of community mental health supports in Australia. If the P-PROM ROCK Program was to be scaled up, these risks and concerns need to be monitored and mitigated to ensure realisation of the full potential of P-PROMs. There are key decisions when scaling up the P-PROM ROCK program about the choice of P-PROM and its role. A generic P-PROM has the advantage of being short and comparable across all disease areas and the clinicians and patients/caregivers in our study highlighted its potential to improve interest in the child’s broader wellbeing. However, a generic P-PROM needs to sit alongside a disease-specific measure when more detail is required about HRQoL in a disease context. Our research highlighted generic and disease-specific P-PROMs as different and potentially complementary.

This study is not without its limitations. Firstly, only two participants in the patient/caregiver group were adolescents, and both took part alongside their caregiver. Although for one patient this made them more comfortable, for the other, the caregiver often spoke on their behalf, limiting adolescent-specific insights. This is a common challenge in paediatric qualitative research where parents play both an advocacy and proxy role for their children. Secondly, the clinicians who took part in this trial did so because they were willing and interested to trial P-PROMS in clinical care, their perspectives may differ to clinicians less interested in P-PROMs. Finally, the researchers conducting the interviews and analysis are invested in the P-PROM ROCK Program, which could affect comments to participants and interpretation of results. Double coding and reflexivity methods were applied to minimise these impacts.

## Conclusion

Making P-PROM programs as engaging, easy and simple as possible is key to the completion and use of P-PROMs in routine paediatric clinical care. The use of a short generic P-PROM, such as the EQ-5D-Y-5L, as well as co-designing simple resources to support the use and integration of P-PROMs into clinical care and clinical systems is essential. With well designed systems and integration, P-PROMs have the potential to ensure specialty clinical care is more holistic, as well as improving communication and satisfaction with care. However, if we ask patients to complete P-PROMs, we need to ensure appropriate resourcing is available to action and respond to P-PROM results. Currently, constrained systems limit this being possible for all patients. A larger and longer trial should be conducted to understand the further potential impacts of P-PROMs identified in this qualitative study, including potential benefits, such as tracking over time, and risks, such as unmet expectations, trauma arising from unaddressed mental health issues and pressure on clinic time.

## Supplementary Information

Below is the link to the electronic supplementary material.

Supplementary file1 (PDF 111 KB)

Supplementary file2 (PDF 176 KB)

## Data Availability

Deidentified data may be made available upon reasonable request.
