# Supplementary material for: Qualitative insights from patient/caregivers, and clinicians on routine use of the EQ-5D-Y-5L in clinical paediatric care—results from a pilot feasibility and acceptability trial
Source: Qual Life Res. 2026 Mar 13;35(4):102. doi: 10.1007/s11136-026-04202-2 (PMC12987837; doi:10.1007/s11136-026-04202-2)
Supplement: Supplementary file 1 — Supplementary file1 (PDF 111 KB) [file 11136_2026_4202_MOESM1_ESM.pdf]

Supplementary Material 1- Caregiver/Child Qualitative Interview Guide

1. Opening Introduction (to be read to participant by research team member):

Thank you for taking the time to participate in this interview. My name is [insert interviewer name], I'm a researcher on the P-PROM ROCK Study.

As you know, as part of the study, we were interested in understanding if asking children and young people, or their caregivers, to fill out a general health tracking questionnaire as part of their hospital appointment was helpful.

During the interview today I will ask you for your views on taking part in the study, and how you found completing the general health tracking questionnaire. I will show you a copy of the general health tracking questionnaire and the resources you received to prompt your memory, as I know it was a while ago that you took part in the study.

If at any stage you'd like to stop, take a break, or don't want to answer any questions, just let me know. Please feel free to bring things up you think might be relevant that we have not asked you directly about. There are no right or wrong answers to questions – just ideas, experiences, and opinions, which are all valuable.

Before we jump into the interview I will just run through a couple of logistical things.

2. Interview Questions

Topic	Checklist – ask if applicable
1. **Experience completing the general health tracking questionnaire (EQ-5D-Y-5L) and additional question about priority for discussion.**	Here is an example of the general health tracking questionnaire you were asked to complete as part of your hospital visit. [Researcher to share screen with example of EQ-5D-Y-5L and extra question].<br><br>How did you find completing the general health tracking questionnaire before your appointment?<br>Prompts:<br>• What did you like?<br>• What didn't you like?

	How did you find completing the additional question, that asked you which of the health areas would be important to discuss in your upcoming appointment?<br>Prompts:<br>• What did you like?<br>• What didn't you like?<br>• Did you feel like you could leave it blank if there was nothing to discuss?
**2. Perceived impact on care**	Do you feel that completing the general health tracking questionnaire was helpful to your/ your child's health care?<br><br>If yes, how?<br><br>If no, why not?
**3. Views on resources and support**	When you completed the general health tracking questionnaire, you received some resources. I will just share my screen with a copy of the resources you received. [Researcher to share screen with copy of resources].<br><br>What did you think of these resources?<br><br>Prompts:<br>• What did you like?<br>• What didn't you like?
**4. Final thoughts.**	Is there anything else you wanted to mention that we may not have already discussed?
