# Supplementary material for: Qualitative insights from patient/caregivers, and clinicians on routine use of the EQ-5D-Y-5L in clinical paediatric care—results from a pilot feasibility and acceptability trial
Source: Qual Life Res. 2026 Mar 13;35(4):102. doi: 10.1007/s11136-026-04202-2 (PMC12987837; doi:10.1007/s11136-026-04202-2)
Supplement: Supplementary file 2 — Supplementary file2 (PDF 176 KB) [file 11136_2026_4202_MOESM2_ESM.pdf]

Supplementary Material 2 - Clinician Focus Group Discussion Guide

The focus groups will be semi-structured to give clinician participants an opportunity to discuss their views and preferences on the feasibility and acceptability of using the generic P-PROM in routine outpatient clinical care.

1. Welcome, introduction of researchers, thanks for participation and background regarding area of research and the focus group
2. Housekeeping, ground rules
    - Reminder that the focus group will be recorded
    - Explain that they can withdraw from the study at any time
    - Explain that if they find anything upsetting or distressing they can send a private message to the facilitator.
    - Establish “ground rules”:
        - Only one person talks at a time.
        - Participants are asked to keep all information discussed confidential. “What is shared in the room stays in the room.”
        - It is important for us to hear everyone’s ideas and opinions. There are no right or wrong answers to questions – just ideas, experiences and opinions, which are all valuable.
3. Participant introductions (warm up to get used to speaking in a group setting)
4. Discussion about what people did and didn’t like. Prompts: EPIC, display, resources, training.
5. Discussion about impact on care.
6. Discussion about mechanisms for being useful or not useful. Prompts: impact on the condition versus things beyond the condition.
7. Discussion about impact on role as clinician.
8. Discussion about harms and risks. Prompts: risks that did or didn’t eventuate.
9. Discussion about ways to improve for future.
10. Final thoughts from participants, summary and thank participants.
